# The Risk of Disease to Great Apes: Simulating Disease Spread in Orang-Utan (*Pongo pygmaeus wurmbii*) and Chimpanzee (*Pan troglodytes schweinfurthii*) Association Networks

**DOI:** 10.1371/journal.pone.0095039

**Published:** 2014-04-16

**Authors:** Charlotte Carne, Stuart Semple, Helen Morrogh-Bernard, Klaus Zuberbühler, Julia Lehmann

**Affiliations:** 1 Centre for Research in Evolutionary and Environmental Anthropology, University of Roehampton, London, United Kingdom; 2 The Orang-utan Tropical Peatland Project, Centre for International Cooperation in Sustainable Management of Tropical Peatland, Universitas Palangka Raya, Palangka Raya, Central Kalimantan, Indonesia; 3 Centre for Research in Animal Behaviour, College of Life and Environmental Sciences, University of Exeter, Exeter, United Kingdom; 4 School of Psychology and Neuroscience, University of St Andrews, St Andrews, Fife, United Kingdom; 5 Cognitive Science Centre, University of Neuchâtel, Neuchâtel, Switzerland; University of Missouri, United States of America

## Abstract

All great ape species are endangered, and infectious diseases are thought to pose a particular threat to their survival. As great ape species vary substantially in social organisation and gregariousness, there are likely to be differences in susceptibility to disease types and spread. Understanding the relation between social variables and disease is therefore crucial for implementing effective conservation measures. Here, we simulate the transmission of a range of diseases in a population of orang-utans in Sabangau Forest (Central Kalimantan) and a community of chimpanzees in Budongo Forest (Uganda), by systematically varying transmission likelihood and probability of subsequent recovery. Both species have fission-fusion social systems, but differ considerably in their level of gregariousness. We used long-term behavioural data to create networks of association patterns on which the spread of different diseases was simulated. We found that chimpanzees were generally far more susceptible to the spread of diseases than orang-utans. When simulating different diseases that varied widely in their probability of transmission and recovery, it was found that the chimpanzee community was widely and strongly affected, while in orang-utans even highly infectious diseases had limited spread. Furthermore, when comparing the observed association network with a mean-field network (equal contact probability between group members), we found no major difference in simulated disease spread, suggesting that patterns of social bonding in orang-utans are not an important determinant of susceptibility to disease. In chimpanzees, the predicted size of the epidemic was smaller on the actual association network than on the mean-field network, indicating that patterns of social bonding have important effects on susceptibility to disease. We conclude that social networks are a potentially powerful tool to model the risk of disease transmission in great apes, and that chimpanzees are particularly threatened by infectious disease outbreaks as a result of their social structure.

## Introduction

Great apes are susceptible to a wide range of diseases, including Ebola [1], polio-like diseases and mange [2], measles and scabies [3], influenza [4], tuberculosis [5] and various respiratory diseases [2], [6]–[9]. Because all apes have a long life history, populations need considerable time to recover from epidemics [9]. Although not all long-term chimpanzee (*Pan troglodytes spp*.) field sites have been affected by lethal epidemics, some have suffered great losses due to diseases. Respiratory epidemics have affected a number of study sites [2], [6]–[9], with indications that some infections have been transmitted from humans [8], [10]. In chimpanzees, morbidity varied from 20 to 98%, with death rates of between 3 and 17% [7], [9]; such disease outbreaks are therefore of great concern to researchers and conservationists. As a response, various study sites have put in place a range of rules to try to prevent disease transmission despite the difficulties of enforcing them [3], [11]. In contrast to chimpanzees, there are no documented large scale epidemics in orang-utans (*Pongo spp.*), although there are reports of disease transmission from humans. For example at Ketambe, Sumatra, an influenza type disease and conjunctivitis have been passed from human caretakers to rehabilitant orang-utans, with the former then passed on to two wild orang-utans [4]. Orang-utan rehabilitation sites often host tourists who, if ill and infectious, pose a serious health risk to the animals [12]. While disease transmission from humans to great apes has become an inherent problem associated with ecotourism and scientific research, natural diseases that affect great apes in the absence of humans will also continue to be a threat [1], [13], [14]. In conclusion, understanding how diseases spread within groups and populations of great apes is of vital importance to implement effective preventative measures and to minimise the risk of losing individuals, and ultimately the species, to diseases.

Patterns of disease spread are influenced by a number of parameters, most importantly by the social organisation of a species and disease-specific parameters, such as transmission mode, infectiousness and time to recovery. For example, data from humans suggest that highly infectious diseases, such as Ebola, measles or influenza, have infectious periods lasting for 10 days, 6–7 days and 2–3 days respectively, while tuberculosis is less infectious but has a longer infectious period [15]–[17]. In order to react and plan adequately it is therefore important to make informed predictions of how different diseases are likely to spread within different social groupings. This type of information could help to identify the most effective strategies for both responding to and preventing epidemics.

So far, virtually no epidemiological models exist for great apes, although social network-based approaches of disease transmission have been used for African buffalo (*Syncerus caffer*) [18], brushtail possums (*Trichosurus vulpecula*) [19] and killer whales (*Orcinus orca*) [20]. For African buffaloes, the model predicted that slowly spreading diseases would affect more individuals than rapidly spreading diseases, as a result of the movement of individuals between groups over time [18]. In possums, contact patterns predicted that bovine tuberculosis would spread within the entire population if more than 8% of contacts led to secondary infections [19], while killer whales were shown to be highly susceptible to disease spread as a result of both the topology of the network and the strength of relationships within it [20]. These models provide first predictions of the way that diseases with different properties would spread within wildlife populations and as such give indications as to which diseases might cause the largest loss of individuals.

In this study, we use epidemiological modelling to explore disease spread in chimpanzees and orang-utans. Both species are characterised by fission-fusion social systems; relationships are fluid, with individuals assembling in temporary parties that regularly change in composition [21], [22]. Within this general classification, orang-utans and chimpanzees lie at opposite ends of the spectrum in terms of gregariousness. Chimpanzees spend a far larger proportion of time in association, while orang-utans spend the majority of their time alone or with dependent offspring [4], [23]–[26]. This difference is likely to affect the risk that disease poses to each species. Traditional disease models are typically based on homogenously mixed populations, in which all individuals are equally likely to interact with all other individuals, so called mean-field models [15], thereby ignoring the details of species-specific social dynamics. More recent models have incorporated the natural heterogeneity of contact patterns using social network analysis. A typical finding is that the topology of the network can have a considerable impact on the predicted disease spread [18], [27]–[29]. For example, simulations of disease transmission in African buffalo indicated a much faster spread of disease on a mean-field network than on actual association networks [18]. Despite the advantages and presumably greater precision in predictions of the social network approach, it has not yet been employed widely in wildlife epidemiological models as it is data intensive and requires detailed behavioural observations.

Here, we used a social network approach to simulate predicted disease spread in wild orang-utans and chimpanzees, in order to assess the threat that disease poses to these species. We focused specifically on diseases that are transmitted through close proximity or direct contact between individuals, such as respiratory diseases. We employed a susceptible-infected-recovered network modelling approach to investigate the potential spread of disease in association networks from a population of 37 orang-utans from the Sabangau forest, Indonesia, and 55 members of a chimpanzee community from Budongo, Uganda. Our aims were (i) to determine the susceptibility of the orang-utan and chimpanzee networks to the spread of diseases with differing infectiousness and probability of recovery, (ii) to compare the association network approach with the more traditional mean-field approach, to determine if the topology of the network impacted predicted disease spread, and (iii) to compare the results between the species to highlight the impact of gregariousness on the threat of disease.

## Methods

### Ethics Statement

Permits and ethical approval for the field studies were obtained from the Indonesian Institute of Sciences and the Ministry of Research and Technology and the Uganda National Council for Science and Technology, the Ugandan Wildlife Authority and the National Forestry Authority.

### Association Data and Network Construction

We constructed networks for both species using association data, i.e. presence in the same party, where a party was defined as all individuals within 50 m of each other. The orang-utan data were collected from 2003–2011 as part of the OuTrop multi-disciplinary research project in collaboration with CIMTROP, in the Natural Laboratory for the Study of Peat Swamp Forests (2°19′S 114°00′E). The population comprised 46 individuals: four adolescent females, 10 adult females, two adolescent males, 16 unflanged males and 14 flanged males. Nine of these orang-utans were never observed in association with other individuals and so were excluded from the analyses, as this study focuses on diseases that are transmitted through close proximity between individuals or through direct individual-to-individual contact. Data were collected during focal follows that lasted for as long as 10 consecutive days. Association data were recorded using instantaneous sampling every five minutes. In total, 165,717 focal scans were recorded.

The chimpanzee data were collected between August 2007 and July 2010 on 55 members of the Sonso community of Budongo Forest: 12 adolescent females, 24 adult females, eight adolescent males and 11 adult males. Data were collected during focal follows and association data recorded using scan samples every 15 minutes. In total, 34,143 focal scans were recorded.

Weighted association networks were constructed from the association data, using Dyadic Association Indices (DAIs) as the weights of the edges:


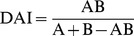


where *A* is the total number of times that *A* was observed, either alone or with other independent individuals, *B* is the total number of times *B* was observed and *AB* is the total number of times that *A* and *B* were observed together. Association indices range from zero to one, with zero indicating that two individuals were never observed together and one indicating that they were always observed together.

### Disease Simulations: Susceptible-infected-recovered Models

We simulated the spread of disease using a susceptible-infected-recovered model. This involved allocating each individual in the network one of three states at all times: susceptible, infected or recovered. The simulation begins with the infection of one individual in the network, patient zero; this individual is selected at random. All other individuals start the simulation as susceptible. The spread of disease from patient zero to its contacts is assumed to be a function of the transmission coefficient β, representing the infectiousness of the disease, and the dyadic association index, representing the probability that a dyad will associate. At each time step, disease spreads from infected to susceptible individuals with a probability that is the product of these two variables. Once infected, individuals recover with a probability γ, the recovery coefficient, and do not return to susceptible status. It is important to note that conceptually, recovered individuals are equivalent to dead individuals. In all cases the individual is removed from the network and can no longer transmit disease. In terms of modelling subsequent disease spread it is consequently not important to distinguish the number of individuals that recover from the number that die and so here both states will be referred to as recovered.

### Simulating the Spread of Different Diseases

We ran all simulations using tnet [30] in R [31]. The simulations were run with a range of values for the transmission and recovery coefficients, to simulate the spread of diseases with differing levels of infectiousness and recovery. We varied the transmission coefficient and the probability of recovery from 0.1 to 1.0 at intervals of 0.1 and simulated the spread of diseases with all 100 combinations of values (i.e. 0.1 and 0.1, 0.1 and 0.2, 0.1 and 0.3 etc.). Each simulation stops when disease has stopped spreading and all infected individuals have recovered. At this stage, the total number of individuals that were infected is calculated to give the final size of the epidemic. For each combination of parameters we ran the simulation 10,000 times and calculated the mean final size of the epidemic.

### The Effect of Network Topology on Predictions

We explored the effect of network topology on the spread of disease by comparing the size of the epidemics predicted on the association networks with the size of the epidemics predicted on mean-field networks, where individuals mix homogeneously. In the mean-field network, all individuals were connected to all others and each dyad associated with an association index that was equivalent to the mean of the association indices in the actual network. This ensured that the overall force of infection in the mean-field model was the same as that in the association network [18]. Again, we varied the transmission and recovery coefficients between 0.1 and 1.0 and tested all 100 combinations of parameters. We simulated the spread of disease on the mean-field networks 10,000 times for each combination of parameters and calculated the final size of the epidemic.

## Results

### The Spread of Disease in the Orang-utan Network

**Figure 1:**
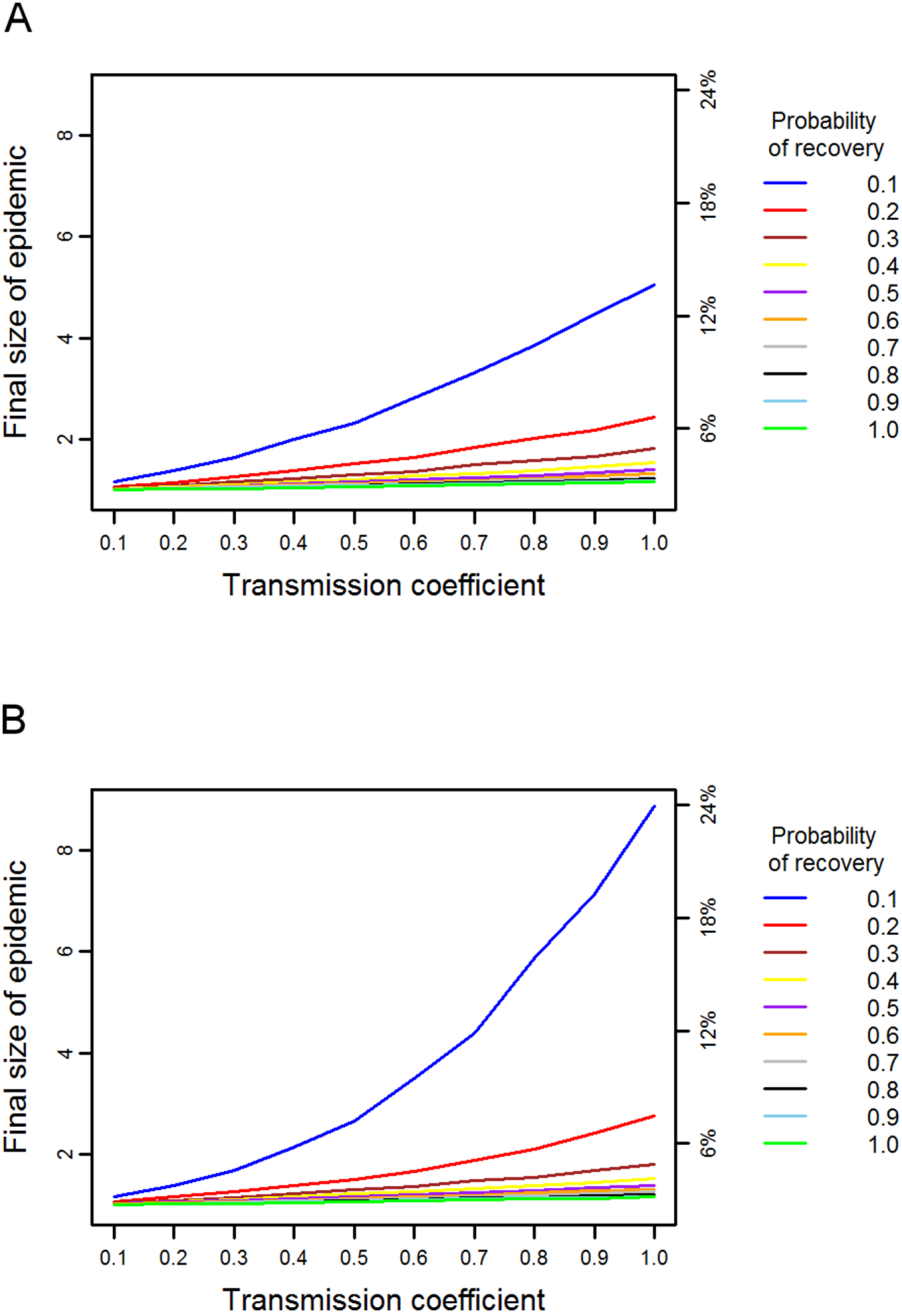
Predicted disease spread in the orangutan network. The final size of the epidemic in terms of absolute size and the percentage of the population, for diseases with different combinations of transmission and recovery probabilities in (a) the orang-utan association network and (b) the mean-field network.

Simulating the spread of diseases with differing transmission coefficients and probabilities of recovery on the orang-utan association network indicated that disease does not spread extensively under any combination of these parameters (Figure 1a). Even with a very low probability of recovery of 0.1 per time step and a very high transmission coefficient of 1.0, on average only five of the orang-utans (ca. 14% of the population) became infected.

The spread of disease across the mean-field network (Figure 1b) was very similar to that across the association network. All combinations of the transmission coefficient and the probability of recovery produced almost identical results on the mean-field network as those on the association network. The only exception was diseases with very low recovery (γ = 0.1) and high infectiousness (β>0.6), but even here, the greatest difference found between the predictions was less than four individuals (ca.10% of the population). Thus, for orang-utans association data appear to be irrelevant to predict the number of individuals infected, regardless of disease type.

### The Spread of Disease in the Chimpanzee Network

**Figure 2:**
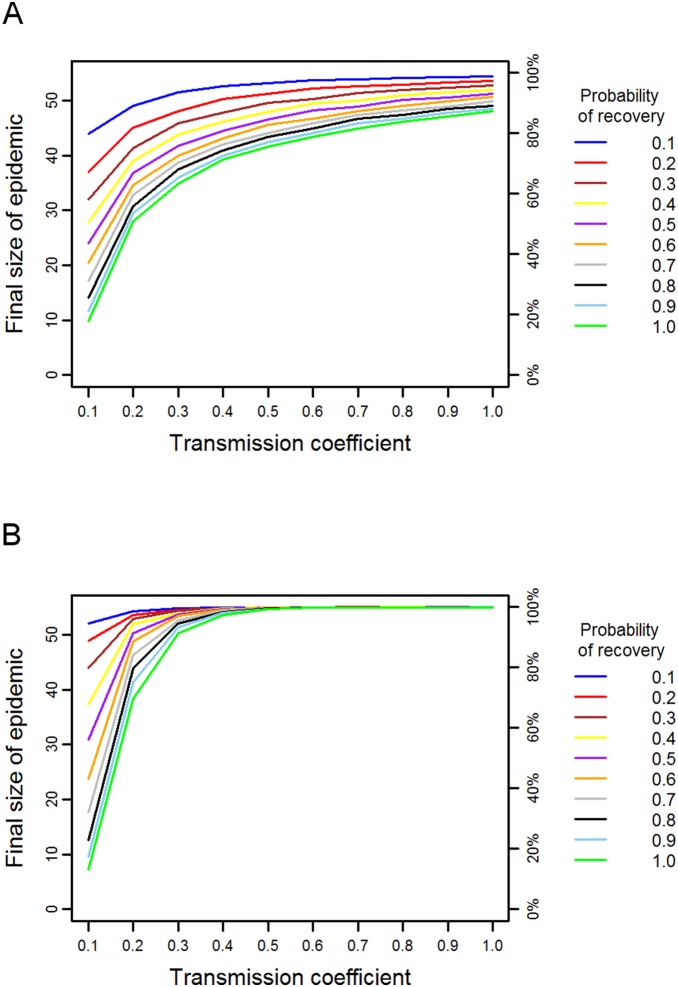
Predicted disease spread in the chimpanzee network. The final size of the epidemic in terms of absolute size and the percentage of the population, for diseases with different combinations of transmission and recovery probabilities in (a) the chimpanzee association network and (b) the mean-field network.

Simulations on the chimpanzee network indicated a much higher degree of vulnerability to disease than predicted for the orang-utan (Figure 2a). Diseases with a high probability of transmission, i.e. highly infectious diseases, spread to almost all members of the network even when combined with a high recovery probability. Indeed, even if recovery was certain at each time step (γ = 1.0), a disease only needed a transmission probability of 0.5 in order to reach over 40 members (73%) of the chimpanzee community on average. Diseases with a low probability of transmission and a high probability of recovery did not spread as much in the network; entering a minimum transmission probability of 0.1 and a maximum recovery probability of 1.0 generated a final epidemic size of 9.76 individuals (17.7% of the community). Increasing the transmission coefficient led to large relative increases in the final size of the epidemic, while increasing the probability of recovery had a smaller effect on total number infected by the epidemic. The chimpanzee network therefore appears to be susceptible to diseases with a range of parameters, but particularly to those with intermediate to high transmission coefficients.

Comparing the spread of disease on the chimpanzee association network with that on the mean-field network produced very different results to those seen in the simulations for orang-utans. Regardless of parameter combinations, the final size of the epidemic on the mean-field network was higher than that on the association network (Figure 2b). Only diseases with very high recovery rates (γ>0.7) and low transmission coefficients (β = 0.1) spread more on the association network than on the mean-field network. Excluding these extreme cases, on average an additional 7.53 chimpanzees, or 14% of the community (range 1–22%), were predicted to become infected on the mean-field network compared to the association network. Incorporating heterogeneity in contact patterns therefore has an important effect on the predicted disease spread.

## Discussion

The spread of a range of diseases with differing infection and recovery parameters was simulated in a community of chimpanzees and a population of orang-utans, to assess the vulnerability of these species to epidemics. While disease was not predicted to spread rapidly or extensively through the orang-utan population, the chimpanzee community was predicted to be extremely vulnerable to disease. Furthermore, the topology of the association network was found to have an important effect on the predictions of disease spread for chimpanzees, but not for orang-utans. It is important to note that the simulations were based on association networks using data collected over nine years for the orang-utans and three years for the chimpanzees. Although this difference prevents any detailed quantitative comparisons being made between the two species, the markedly different overall patterns that emerged highlight differences in how disease is likely to spread in each species. Overall, our results are relevant for the planning of conservation initiatives and disease prevention measures. While disease risk should not be ignored for orang-utans, infectious diseases represent a particularly major threat for wild chimpanzees and effective measures to prevent disease from entering communities should therefore be implemented, especially in habituated populations.

As with all modelling approaches, there are a number of simplifications/generalisations that needed to be made, which are important to discuss. The definition of social contact employed here may have important influences on the inferences that can be drawn. Both orang-utans and chimpanzees were said to be associating if they were within 50 metres of one another (a commonly used definition by field workers). Although for much of the measured association time individuals will in fact be in much closer proximity than the 50 metre cut-off distance, in reality many diseases require very close contact for transmission to take place [32]. Examples include sexually transmitted diseases and those caused by parasites, which are also further complicated by stages of the life cycle spent outside the host and the number of parasites infecting each host [15], [33]. In addition, many diseases are spread through the faecal-oral route where the link between association and transmission is less clear; for example, viruses causing diseases such as polio can survive for several months in the soil [32] and so may be transmitted between individuals that have never been in proximity. In some cases, using contact networks instead of association networks might be more appropriate, and similar models to those used here could be run on these other types of networks. Our results do, however, provide a general model of the spread of respiratory diseases, which are both relatively common and extremely threatening to great apes [2], [9]. Improving our understanding of the spread of respiratory disease is important for these species, especially in regards to the appropriate management of both research and ecotourism sites.

The simulations used here were based on static association networks, as opposed to dynamic networks which include temporal changes in contact patterns [34]. Static networks are assumed to provide an accurate representation of the relationships between individuals in the population or community, and hence of the overall social organisation [20]. Although our networks may be biased towards core individuals that are sampled more often, a model run on a static network is assumed to provide an indication of the way in which disease would be expected to spread on average, based on the overall structure of the society. This may be misleading as it fails to account for short-term relationships that change as a result of ecology or demography, which could have an important effect on the pattern of disease transmission [35]. Therefore, it may be useful to also analyse dynamic networks, in which relationships vary over shorter periods of time and disease spreads in accordance with the contact patterns present during that particular time period [18]. However, this would require a large amount of data collected over short periods of time to ensure that relationships are adequately sampled. As such extensive databases are rare, most network models to date have been static [36]. The orang-utan social system in particular, with individuals dispersed over large areas and spending considerable amounts of time alone, would be extremely difficult to sample sufficiently to create a reliable dynamic model. Using data collected over a long period of time ensures that rare relationships are included, producing a more accurate representation of the general social structure (and hence patterns of disease spread) [37].

### The Impact of Gregariousness on Predicted Disease Spread

The disease simulations described in this paper, using a range of transmission and recovery parameters, provide clear evidence of the impact that differences in gregariousness between the orang-utans and chimpanzees have on predicted vulnerability to disease spread. Even diseases with very high infectiousness and slow recovery did not infect more than five of the 37 orang-utans (14%). A highly infectious disease associated with low recovery is, however, clearly a worst-case scenario, although it should be emphasised that the loss of five individuals could have an important impact on the population. The chimpanzee network, by contrast, was highly susceptible to diseases with a broad range of transmission and recovery parameters, particularly those with medium to high transmission coefficients. At a number of study sites, respiratory epidemics have indeed been shown to affect the majority of chimpanzees [8], [38]. This demonstrates that chimpanzee communities are likely to be extremely susceptible to even moderately contagious diseases, while very contagious diseases such as measles and pertussis [39] may have catastrophic consequences. Even diseases with low infectivity and rapid recovery, a best case scenario in terms of disease parameters, spread to a high number of chimpanzees (18%). Chimpanzees are clearly susceptible to disease spread, and the extent of this vulnerability, encompassing diseases varying widely in transmission and recovery parameters, is a serious conservation concern. The spread of diseases from humans has already been implicated in a number of epidemics in chimpanzees [2], [7] and mortality from disease is often high [9]. Ecotourism has many associated benefits, such as providing finances and local support for the conservation of great apes [32], and so it is not practical to recommend the complete cessation of great ape tourism. However, the speed with which diseases can spread between chimpanzees is a clear warning that strict hygiene measures must be enforced to prevent the introduction of disease into the chimpanzee communities.

The relative lack of disease spread predicted among orang-utans, particularly in comparison to the chimpanzee, suggests that orang-utans are unlikely to be regularly affected by infectious disease, as a result of their social system. Further studies should investigate the extent to which this finding applies to orang-utans in rehabilitation centres, which live at much higher densities than those in the wild [40]. In these conditions, it is possible that orang-utan social structure is in fact closer to that of the chimpanzee than wild orang-utans, leading to a much higher risk of disease spread.

### The Impact of Network Topology on Predicted Disease Spread

Although models with the most realistic parameters should produce the most accurate predictions, it is often impossible or excessively time consuming (in terms of data collection) to obtain sufficiently detailed data. For these reasons, in network epidemiological models, mean-field networks are often used instead of actual association or interaction networks. Our results show that for orang-utans, the mean-field network produced similar results to those from the association network for almost all combinations of parameters tested. This indicates that the fine-grained structure of the orang-utan network has little impact on predicted transmission patterns, with the exception of diseases with long recovery times and high infectiousness. It seems likely that the low levels of association between orang-utans in the network limit the spread of disease, regardless of the exact topology of the network. Consequently, the predictions produced here are likely to be widely applicable to other populations of Bornean orang-utans, which are known to spend a comparable amount of time alone as the population studied here [24], [25]. Orang-utans in Sumatra have been found to be more gregarious than those in Borneo [4], [22] and so these populations may face a somewhat higher risk of disease transmission, but this is still likely to be lower than that found for the chimpanzee.

The results from the mean-field network for the chimpanzee provide support for the value of using an actual association network approach in disease simulations for this species, as the predicted final sizes of the epidemics on the mean-field network differed considerably from those on the association networks, in most cases being greater. It is likely that there is a threshold level of association above which it becomes useful to incorporate association data. Without data from a wider variety of social systems it is difficult to estimate where this threshold may lie; however, it is clear that for highly gregarious species such as chimpanzees, the inclusion of (ideally fine-grained) association data can have important effects on predictions.

### Wider Implications of Modelling Results: Information Flow and Culture

The models presented here to assess disease spread dynamics can also be interpreted as models for the spread of social information and the evolution of culture [41]. The results can be directly interpreted in terms of the ease of information flow, and suggest that information is likely to flow faster and to a greater number of individuals among chimpanzees than orang-utans. This adds to the current debate about the spread and acquisition of traditions in chimpanzees and orang-utans. Both species have been shown to exhibit a range of behaviours, such as using tools to obtain social insects or using leaves to collect drinking water, that could be classed as traditions or culture [42], [43]. Geographical variation in these behaviours has not been explained by genetic or ecological differences and has therefore been attributed to local innovations and social learning [44], [45]. Observations show that chimpanzees have a larger cultural repertoire than orang-utans, and it has been suggested that this may result from greater opportunities for social learning as a consequence of higher overall gregariousness [42], [43], [45]. This hypothesis seems to be supported by our findings, although the spread of cultural behaviours might differ slightly from that of disease in that individuals are unlikely to forget a learned behaviour (i.e. recover). However, even at very low recovery rates (which would indicate a very high probability of retaining a behaviour) disease did not spread widely between orang-utans, despite the fact that nine years of data were used to compile the association network. This implies that there are indeed limited opportunities for the transmission of social information between orang-utans, which may help to explain why they are characterised by fewer traditions.

## Conclusion

The results of this study indicate that while orang-utans seem to be at low risk of suffering disease epidemics, for chimpanzees disease represents a major threat. Once a single chimpanzee is exposed to a contagious pathogen, our model predicts rapid and extensive spread within the community. This emphasises the importance of this issue for the future conservation of the chimpanzee, and highlights the value of modelling approaches to the study of wildlife diseases.
